# Functional Characterization of the Nemertide α Family of Peptide Toxins

**DOI:** 10.1021/acs.jnatprod.1c00104

**Published:** 2021-08-16

**Authors:** Erik Jacobsson, Steve Peigneur, Håkan S. Andersson, Quentin Laborde, Malin Strand, Jan Tytgat, Ulf Göransson

**Affiliations:** † Pharmacognosy, Department of Pharmaceutical Biosciences, Biomedical Center, Uppsala University, Box 591, SE-751 24, Uppsala, Sweden; ‡ Toxicology & Pharmacology, University of Leuven (KU Leuven), O&N 2, PO Box 992, Herestraat 49, 3000, Leuven, Belgium; § Department of Medical Biochemistry and Biophysics, Karolinska Institutet, 17177 Stockholm, Sweden; ⊥ Swedish Species Information Centre, Swedish University of Agricultural Sciences, 75007 Uppsala, Sweden

## Abstract

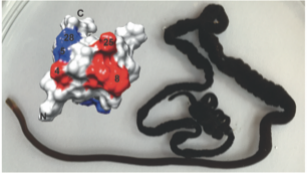

Peptide toxins find use in medicine, biotechnology, and agriculture. They are exploited as pharmaceutical tools, particularly for the investigation of ion channels. Here, we report the synthesis and activity of a novel family of peptide toxins: the cystine-knotted α nemertides. Following the prototypic α-1 and -2 (**1** and **2**), six more nemertides were discovered by mining of available nemertean transcriptomes. Here, we describe their synthesis using solid phase peptide chemistry and their oxidative folding by using an improved protocol. Nemertides α-2 to α-7 (**2**–**7**) were produced to characterize their effect on voltage-gated sodium channels (*Blatella germanica* BgNa_V_1 and mammalian Na_V_s1.1–1.8). In addition, ion channel activities were matched to *in vivo* tests using an *Artemia* microwell assay. Although nemertides demonstrate high sequence similarity, they display variability in activity on the tested Na_V_s. The nemertides are all highly toxic to *Artemia*, with EC_50_ values in the sub-low micromolar range, and all manifest preference for the insect BgNa_V_1 channel. Structure–activity relationship analysis revealed key residues for Na_V_-subtype selectivity. Combined with low EC_50_ values (e.g., Na_V_1.1: 7.9 nM (α-6); Na_V_1.3: 9.4 nM (α-5); Na_V_1.4: 14.6 nM (α-4)) this underscores the potential utility of α-nemertides for rational optimization to improve selectivity.

Animal peptide toxins associated with defense and predation have long been exploited for medicinal, agricultural, and biotechnological applications.^1,2^ Most of those peptides originate from a limited number of taxa, including cone snails, scorpions, spiders, and snakes. However, many more animals make use of such compounds, and it is clear that the study of toxins from carefully selected neglected taxa yield new compounds with potent and interesting effects.^3^ In the current work, we characterize the effects of a novel family of peptide toxins, the α-nemertides, as modulators of voltage-gated sodium channels (Na_V_) and on *Artemia salina*. These peptide toxins were discovered from one such overlooked taxon: nemertean worms.

The phylum Nemertea comprises approximately 1350 valid species worldwide.^4^ The majority of species are found in marine environments, though some are freshwater dwelling (22 species)^5^ and a few are terrestrial.^6^ Most of the known species are predators or both predators and scavengers, and they use their eversible proboscis to hunt for prey. Their secretion is known to contain both proteinaceous and low molecular weight toxins (recently reviewed)^7^ and include, for example, peptide neurotoxins.^8,9^ Lately, transcriptomic and genomic studies have revealed additional putative protein toxins in nemertean worms, including possibly hemolytic ion channel modulators (Na^+^, K^+^, Ca^2+^) and serine protease inhibitors.^8,10,11^

Recently, we described the discovery of the prototypic member of a family of peptide toxins, nemertide α-1 (**1**), from the epidermal mucus of the nemertean worm *Lineus longissimus*.^8^ We showed that this conspicuous marine worm also expressed the homologous nemertide α-2 (**2**), together with a larger type of nemertides, called β-nemertides, on the peptide level. By mining the nemertean transcriptomes available at the time, seven full-length α-nemertides (α-1–7; **1**–**7**) and one truncated (α-8) one were identified. All these peptides are concentrated to the Lineidean genera of nemerteans, Figure 1.

**Figure 1:**
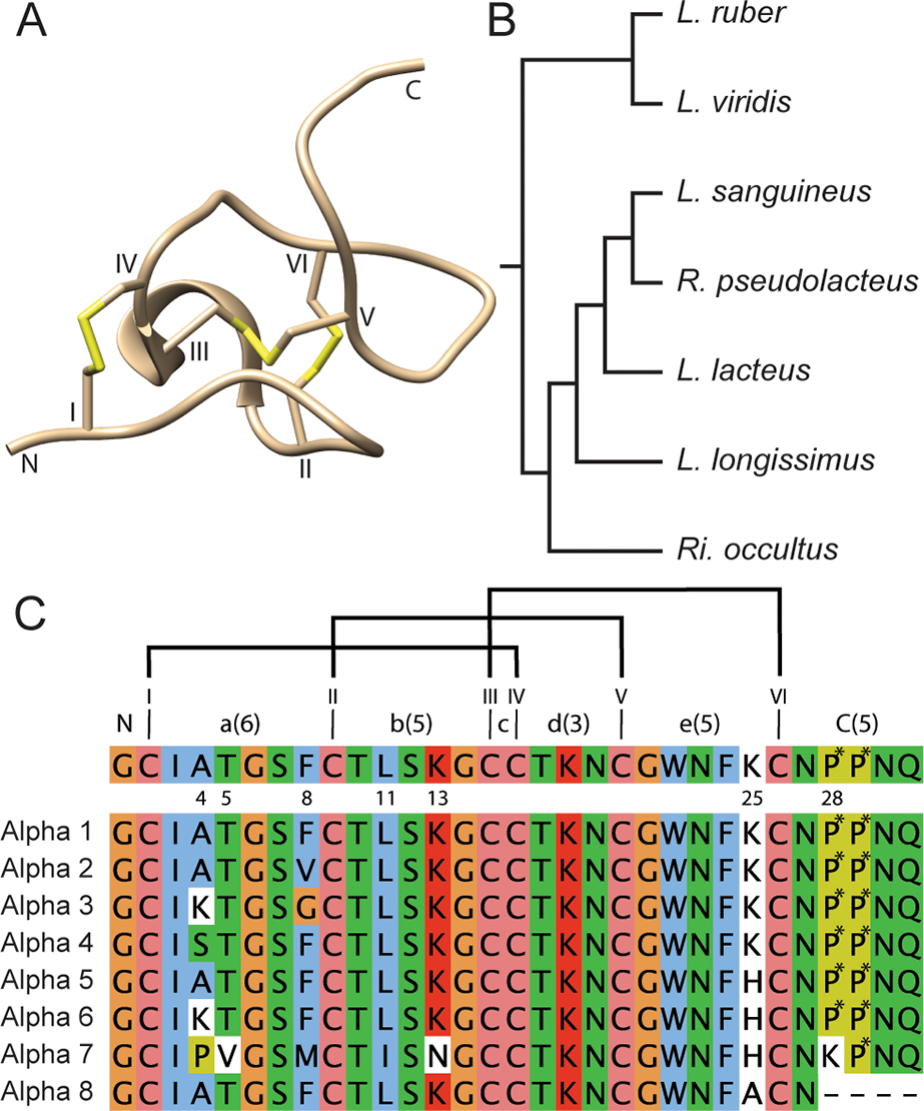
Nemertide structure, sources, and sequences. (A) Solution NMR structure of nemertide α **1** (PDB id 6ENA). The three disulfide bonds at the core of the molecule are numbered (in Roman numerals) and marked in yellow. Numerals mark the loops. (B) Schematic phylogenetic tree of α nemertide expressing species identified on the transcriptomic level. The tree is redrawn from Ament-Velásques et al. 2016.^12^ *: No α nemertide was found in *L. viridis*. (C) Top: consensus α nemertide sequence with the variable positions marked as numbers. Roman numerals mark cysteines, and the ICK disulfide connectivity (I–IV, II–V, III–VI) is displayed with black bars. Loop number in numerals; number of residues in parentheses. Bottom: Sequence alignment of nemertides, including the truncated α-8. P*: hydroxyproline (Hyp). With the exception of **1** and **2**, the α nemertides have so far been identified only in transcriptomic data from the marine Lineideae family of nemertean worms (genus: *Lineus*: 4 sp., *Ramphogordius*: 1 sp., and a partial sequence in *Riseriellus*: 1 sp.) The partial contig of 8 from the *R. occultus* transcriptome was omitted from this study since the full C-terminal sequence was unknown.

Nemertide α-1 (**1**) contains three disulfide bonds and folds into the inhibitory cystine knot (ICK) motif. The structure is compact, consisting of a series of turns and one stretch of secondary structure: a short α-helix in loop 2, Figure 1A. Residues Phe8, Trp22, and Phe24 form an aromatic patch on one side of the molecule.^8^ Sequence similarity is high in the family; for example **2**–**6** differ by only one or two substitutions from **1**, and three-dimensional structures can be assumed to be highly similar, Figure 1. But what differences do these variations confer in activity?

The functional characterization of nemertean peptide and protein toxins is limited so far. Cytolytic and hemolytic effects have been reported for A-cytolysin^13^ and parborlysin proteins,^14,15^ and the 55-residue-long and helical neurotoxin B-IV^16^ and nemertide α-1 (**1**) are paralytic and lethal to crustaceans in sub-nmol/kg doses when injected.^8,16−18^ Mechanism of action has only been shown for α-1, which binds to Na_V_ channels with high affinity and selectivity,^8^ although sodium ion channels have also been suggested to be the target for neurotoxin B-IV.^16^ Nemertide α-1 (**1**) was found to be exceptionally toxic to green crabs (*Carcinus maenas*, lethal at doses above ∼300 pmol/kg) and to cockroaches (*Blaptica dubia*, lethal at 2 nmol/kg). Furthermore, the toxin was shown to modulate insect Na_V_s at low nanomolar concentrations and mammalian Na_V_s in the μM range,^8^ suggesting a possible use for these peptides as bioinsecticides.^19^ This observation is supported by a recent study where pro-alpha-1 was expressed and tested against a selection of insects, demonstrating oral toxicity to aphids and brassica moths.^20^

In the current work, we report the synthesis, oxidative folding, and functional characterization of native members of the α-nemertide family of peptide toxins. All peptides show activity on *Artemia salina*, and their activity on voltage-gated sodium channels of both invertebrate and vertebrate origin reveals selectivity as well as structure–activity relationships.

## Results and Discussion

### Synthesis and Folding of Nemertides

Only two of the α-nemertide toxins (**1**, **2**) have been detected as peptides from natural sources, whereas the discovery of the other five full-length peptides is the result of transcriptome mining. Previously,^8^ we showed that at least **1** is amenable for peptide synthesis: here we demonstrate that the full family can be made using FMOC-based solid phase peptide synthesis (SPPS), with good yields and purity. All α-nemertides were considered to contain Hyp residues in the C-terminus based on the native **1** and **2**.^8^

In analogy with our previous work, **2** was assembled on HMPA resin using automated SPPS on a CEM Liberty1 microwave-assisted synthesizer.^8^ Although this high-swelling resin generally results in good yields, it comes with the practical problem of sometimes blocking drain tubing of the reaction vessel, resulting in stops in the sequence of reactions as well as high maintenance load. Nemertides **3**–**6** were therefore assembled on 2-chlorotrityl resin (Iris Biotech Marktredwitz, Germany), and **7** was purchased from GenScript (Piscataway, NJ, USA) in reduced form. Yields and purities were similar for **3** and **4** compared to the previous synthesis of **1**,^8^ with the target peptides as the major product as judged by HPLC-UV. The synthesis of **5** and **6** gave a higher degree of impurities after cleavage.

The protocol for oxidative folding was substantially improved from previous work.^8^ Notably, extra caution was taken to completely dissolve peptides first in water, followed by addition of DMSO prior to mixing into the folding buffer. No precipitation and/or aggregation of peptides were observed using this procedure for any peptide. We presume that the overall fold of peptides and disulfide connectivity are the same within this peptide family and that all peptides follow the same folding pattern as **1** and **2**. The presumption that peptides **3**–**7** have the overall structure in common with **1** and **2** is further supported by their bioactivity as described below. Then, all correctly folded nemertides **1**–**6** were distinguished from misfolded variants by eluting as a distinct peak late in the chromatogram. Only **7** displayed a more complex folding pattern, where the main product required further purification (using repetitive RP-HPLC).

**Figure 2:**
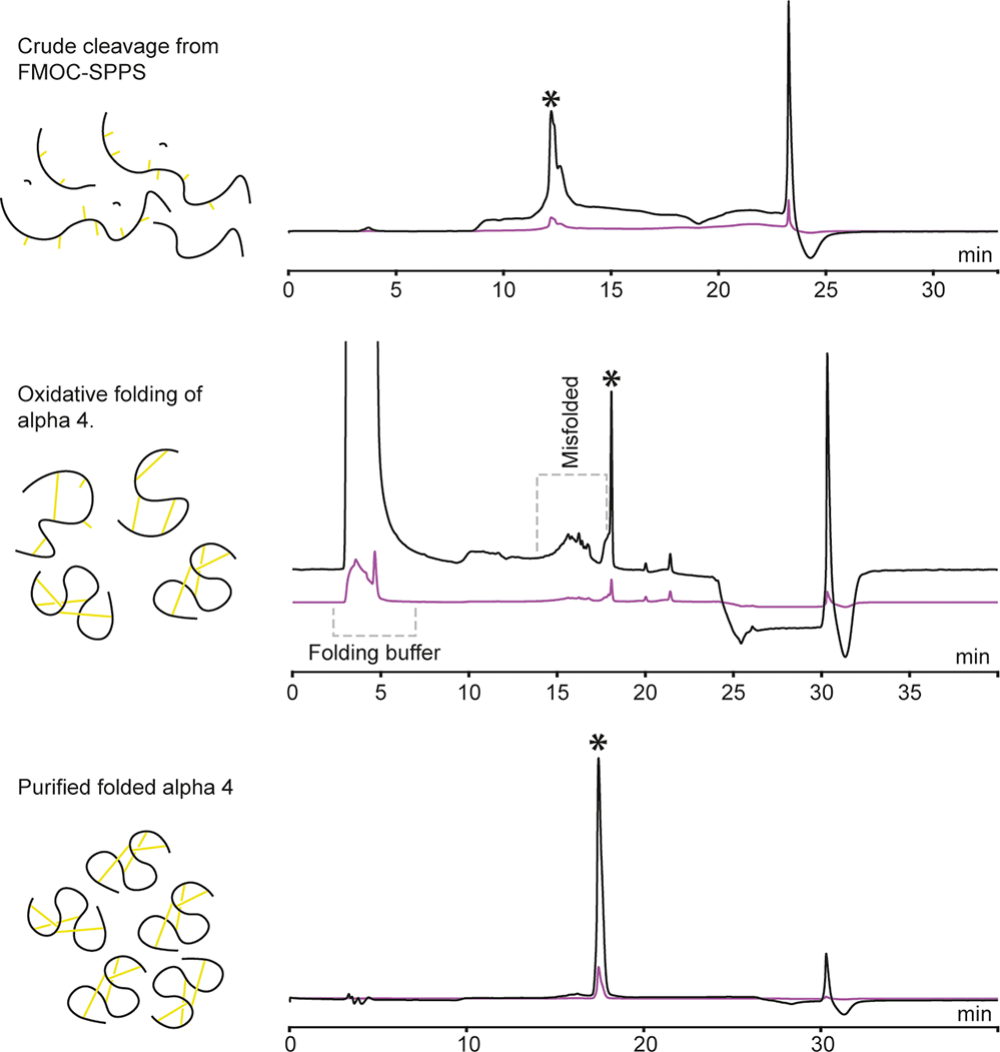
Analytical HPLC-UV traces from synthesis, folding, and final purification of nemertide α **4**. To the left, schematic view of the composition of peptide content. Top: Trace from cleavage of **4** from resin. Middle: Trace from oxidative folding after 15 h. Bottom: Pure folded **4**. *Product wanted in each step. Note that the chromatographic systems differ for the different chromatograms, hence the drift in retention time for the folded **4**. The peaks at ∼24 (top) and 30 min (middle and bottom) are connected to the gradient.

All folded nemertides were purified to >95% as judged by HPLC-UV (215 nm), Figure S1. Typical HPLC-UV chromatograms for crude peptide (cleaved), folding mixture after 15 h, and purified folded peptide are shown in Figure 2. Identities of the purified peaks were analyzed using UPLC-QToF. Expected monoisotopic masses were calculated and compared to the experimental masses obtained from deconvolution of the 4z ions; all experimental masses show errors of less than 10 ppm (Table 1).

**Table 1:** Theoretical and Experimental Molecular Mass Values for α **2**–**7**^a^

	expected (Da)	experimental (Da)	delta (Da)
**2**	3259.3415	3259.3607	0.0192
**3**	3274.3523	3274.3783	0.0260
**4**	3323.3363	3323.3683	0.0320
**5**	3316.3055	3316.3371	0.0316
**6**	3373.3631	3373.3635	0.0004
**7^b^**	3328.7969	3328.35	0.45

a Theoretical and experimentally determined deconvoluted (4z) masses for α **2**–**6**. The 4z ion was chosen since it falls within the calibrated mass range for the QToF used. All values for **2**–**6** are within 10 ppm of the expected masses.

b For **7**, only low-resolution MS (LCQ deca) was available; the convoluted mass was calculated for the doubly charged average mass ion.

### Small Sequence Differences Modulate Physicochemical Properties of Nemertide α Peptides

**Figure 3:**
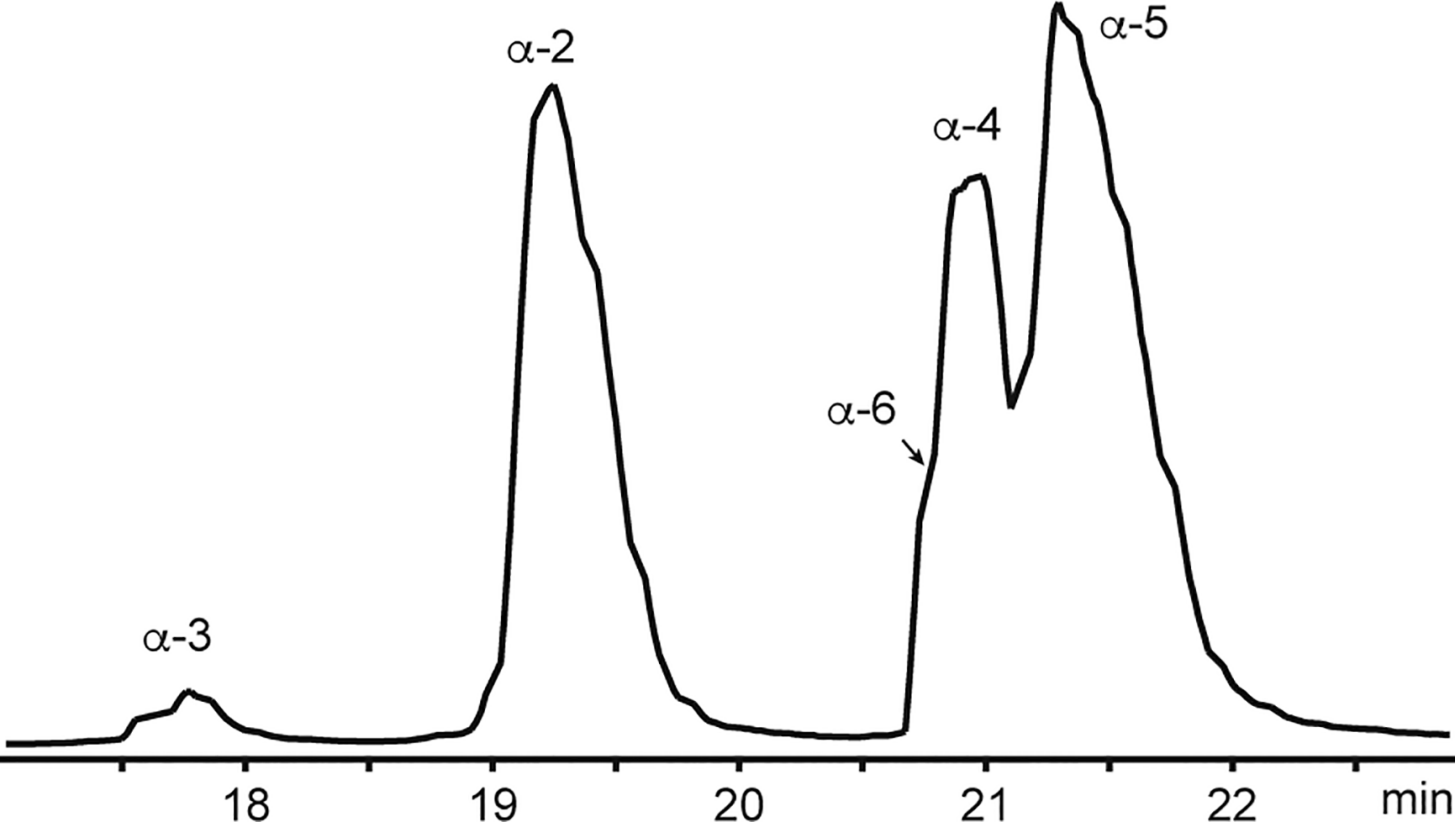
Base peak intensity chromatogram of a mixture of the nemertides **2**–**6**. **3** elutes at 17.7 min, **2** elutes at 19.3 min, and **6** and **4** elute at 20.9–21.0 min. **5** elutes at 21.5 min. The time axis is truncated to show 17 through 23 min. Nemertide α**-**1 was not included in this experiment, but elutes after **2** in a similar system.^8^

Nemertides **2**–**6** were co-injected on the UPLC-QToF (using a C18 column) to evaluate the hydrophilicity. Peptide **7** was not evaluated in these experiments due to its limited availability. All five toxins (**2**–**6**) eluted between 17 and 22 min in the system used, in the elution order **3**, **2**, **6/4**, and **5** (Figure 3). The order of elution reflects changes in the aromatic patch comprising residues in positions 8, 22, and 24; the early eluting **2** and **3** lack an aromatic amino acid in position 8. Furthermore, peptide **3** contains a basic residue Lys4, explaining the early elution at low pH. The elution order of **4**, **5**, and **6** may also be explained by residues in position 4: **6** has a Lys and elutes first of the three, shortly followed by **4** with a hydroxyl-containing Ser in that position. Nemertide α-5 elutes last, with an aliphatic Ala at position 4. The basic Lys/His25 does not seem to influence the retention order on the column.

### Activity on Brine Shrimps Distinguishes Two Groups of α-Nemertides

The clear and rapid effect of toxins on green crabs (*C. maenas*) was a key to their discovery; however the use of these relatively large crustaceans is highly dependent on availability and season. We therefore selected an alternative *in vivo* assay based on the brine shrimp, *Artemia salina*, as a substitute. *Artemia* are small in size, and the assay can be performed in a microwell format using minimal amounts of toxin.^21^ The readout of the assay was the lethality after 24 h, and larvae were not followed over time. However, at the highest concentrations effects could be observed within minutes, with larvae going into a convulsive state followed by death.

**Figure 4:**
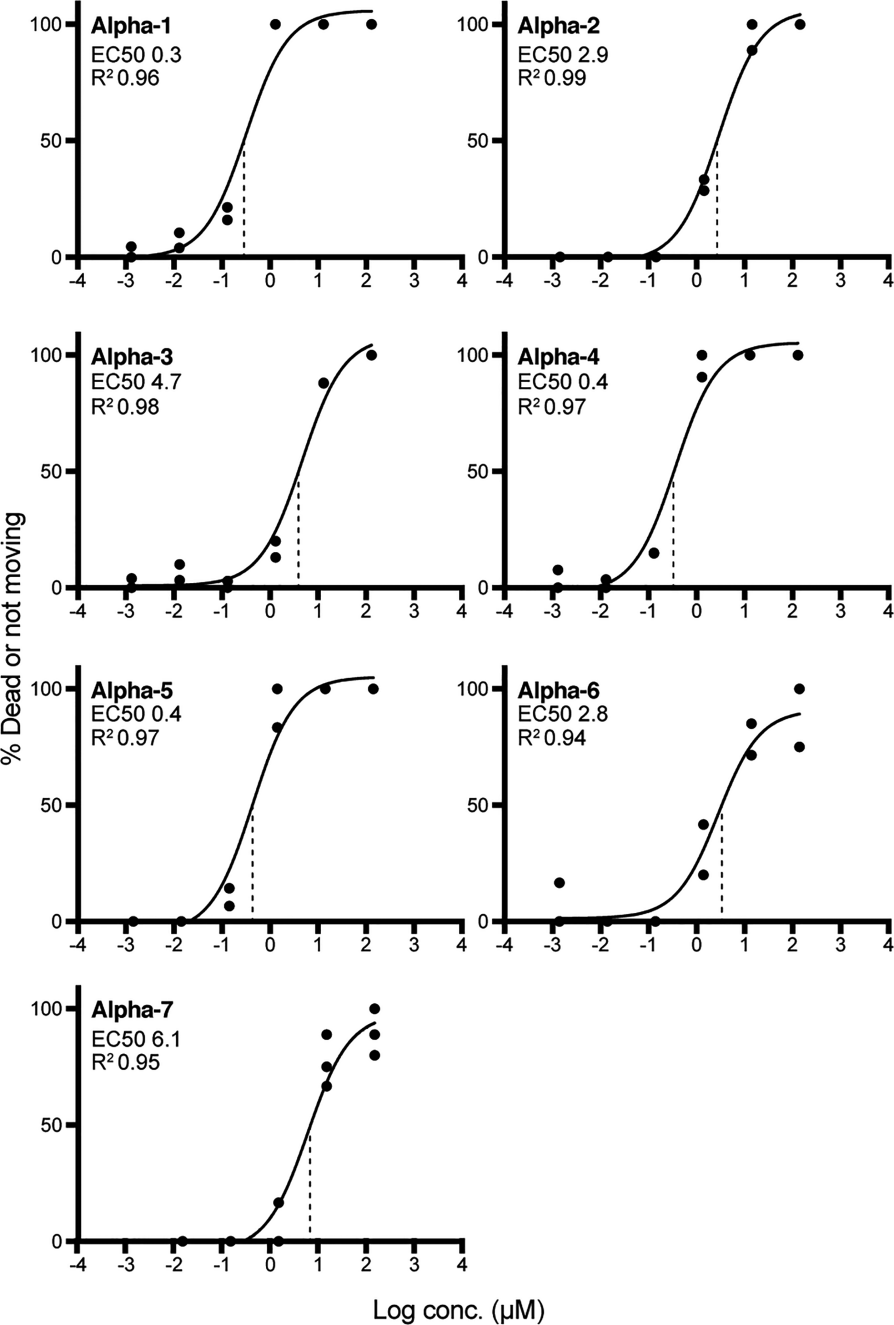
Effect of α-nemertides in the *Artemia* microwell assay. **1**, **4**, and **5** have EC_50_ values in the range 0.3–0.4 μM. **2**, **3**, **6**, and **7** have average EC_50_ values in the range 2.8–6.1 μM. All experiments were performed in duplicate, except **7**, which was done in triplicate. All data points are shown in the graphs (dots), with average fitted values plotted as a line. Vertical dotted lines display the EC_50_s.

All tested α-nemertides (**1**–**7**) exhibit high toxicity to *Artemia*, with EC_50_ values in the sub to low μM range. Furthermore, the activity distinguishes the tested α-nemertides into two groups: **1**, **4**, and **5** display EC_50_ values in the sub μM range (0.3, 0.4, and 0.4 μM, respectively), whereas **2**, **3**, **6**, and **7** have EC_50_ values 1 order of magnitude higher (2.9, 4.7, 2.8, and 6.1 μM, respectively). EC_50_ curves are shown in Figure 4.

### α-Nemertides Show Potent and Differing Effects on Na_V_ Channels

All peptides (**1**–**7**) display potent activity on Na_V_ channels, and current results demonstrate structure–activity relationships. They all show the highest activity on insect BgNa_V_1: **1** and **4**–**7** display EC_50_ values in the low nM range (2.6–11.1 nM), whereas **2** and **3** are approximately one order of magnitude less active. The most active of them all is **6**. EC_50_ values of all peptides against all ion channels tested are shown in Table 2. To simplify comparison, EC_50_ values have been normalized to the most active peptide in Supplementary Table 1 and their activity on BgNa_V_1.

**Table 2:** EC_50_ Values (nM) of **1**–**7** in Na_V_1.1–1.8 and BgNav1^a^

	BgNav1	Nav1.1	Nav1.2	Nav1.3	Nav1.4	Nav1.5	Nav1.6	Nav1.7	Nav1.8
α-1	8.6 ± 2.9	124.1 ± 28.7	359.6 ± 89.8	135.4 ± 76.3	145.5 ± 57.5	138.3 ± 25.5	240.4 ± 22.3	76.5 ± 33.9	n.a.
α-2	87.2 ± 10.5	125.8 ± 43.6	97.9 ± 23.2	127.7 ± 44.5	1150.3 ± 217.8	149.2 ± 89.1	1361.8 ± 115.2	1296.7 ± 232.4	n.a.
α-3	97.5 ± 15.6	125.8 ± 43.6	137.8 ± 36.5	138.9 ± 63.2	150.2 ± 72.7	108.4 ± 5.9	92.8 ± 11.2	102.2 ± 5.8	n.a.
α-4	11.1 ± 1.6	92.0 ± 38.8	134.2 ± 34.0	12.9 ± 3.2	14.6 ± 3.6	27.8 ± 4.3	123.6 ± 29.7	80.5 ± 28.3	n.a.
α-5	7.8 ± 3,2	102.1 ± 50.2	156.1 ± 10.5	9.4 ± 3.7	15.4 ± 5.4	132.7 ± 21.8	66.9 ± 8.2	73.0 ± 14.1	n.a.
α-6	2.6 ± 0.3	7.9 ± 1.3	24.3 ± 2.3	105.6 ± 54.9	46.4 ± 7.2	215.2 ± 63.6	36.3 ± 4.3	97.2 ± 8.3	n.a.
α-7	9.5 ± 1.2	171.5 ± 61.6	50.4 ± 16.1	170.2 ± 39.5	810.6 ± 130.4	155.6 ± 18.3	147.6 ± 53.6	129.0 ± 24.6	n.a.

a All experiments were run in triplicate.

b n.a.: not active.

Nemertide α-6 (**6**) also displays the most potent activity of all peptides on mammalian ion channels, with an EC_50_ of 7.9 nM on Nav1.1. This is one order of magnitude higher activity than the effect observed for any other toxin: **1**–**5** and **7** has EC_50_ values in the range of 92–125.8 nM on Nav1.1. Nemertide α-6 (**6**) differs from the other α-nemertides by having the combination of Lys at position 4 and Phe at position 8. It is also the most potent peptide (EC_50_ 24.3 nM) on Nav1.2. Notably, the prototypic α-1 (**1**) is the least active peptide on this particular ion channel (EC_50_ 359.6 nM), and this is also the highest EC_50_ value for **1** on all of the tested Na_V_s.

On the Na_V_1.3 channel, **4** and **5** exhibit substantially higher activity than other peptides. These two peptides also show high potency against Na_V_1.4, but notably the effect of **5** is one order of magnitude lower than that of **4** to Na_V_1.5.

The largest differences between peptides on any ion channel are seen for Na_V_1.4, ranging from the EC_50_ of **4** at 14.6 nM to **2** with an EC_50_ of 1150.3 nM. In contrast, the differences in activity are smallest on Na_V_1.5. On Na_V_1.6, **2** exhibits an EC_50_ (1361.8 nM) one order of magnitude higher than **1**, **4**, and **7** and 2 orders of magnitude higher than **3**, **5**, and **6**. Nemertide **6** is the most potent, with an EC_50_ of 36.3 nM. On Na_V_1.7, **2** is the least active toxin by far, with an EC_50_ value of 1296.7 nM. All other α-nemertides (**1**, **3**, **4**, **6**, **7**) exhibit comparable EC_50_ values on Na_V_1.7.

### Sequence, Structure, and Activity of Nemertean α Peptide Toxins

**Figure 5:**
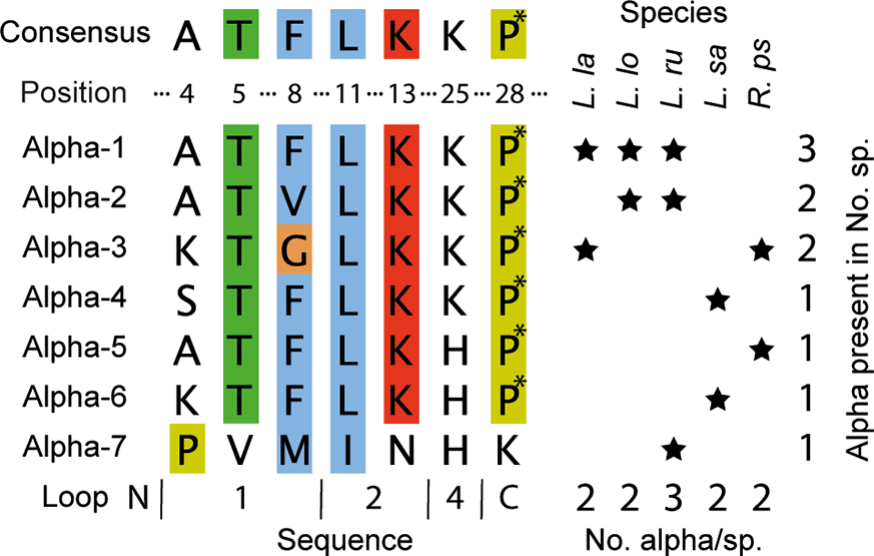
Nemertide α sequence variation and species. Consensus sequence alignment of variable positions. Alignment of the variable positions (positions 4, 5, 8, 11, 13, 25, and 28) of α-nemertides. Species where α-nemertides are found: *L. la*, *L. lacteus*; *L. lo*, *L. longissimus*; *L. ru*, *L. ruber*; *L. sa*, *L. sanguiens*; *R. ps*, *R. pseudolacteus*. **1** is found in three species, and **2** and **3** are found in two species each. **4**–**7** are found in one species each. All investigated species contain two α-nemertide sequences, except *L. ruber*, which harbors three. P*, hydroxyproline.

**Figure 6:**
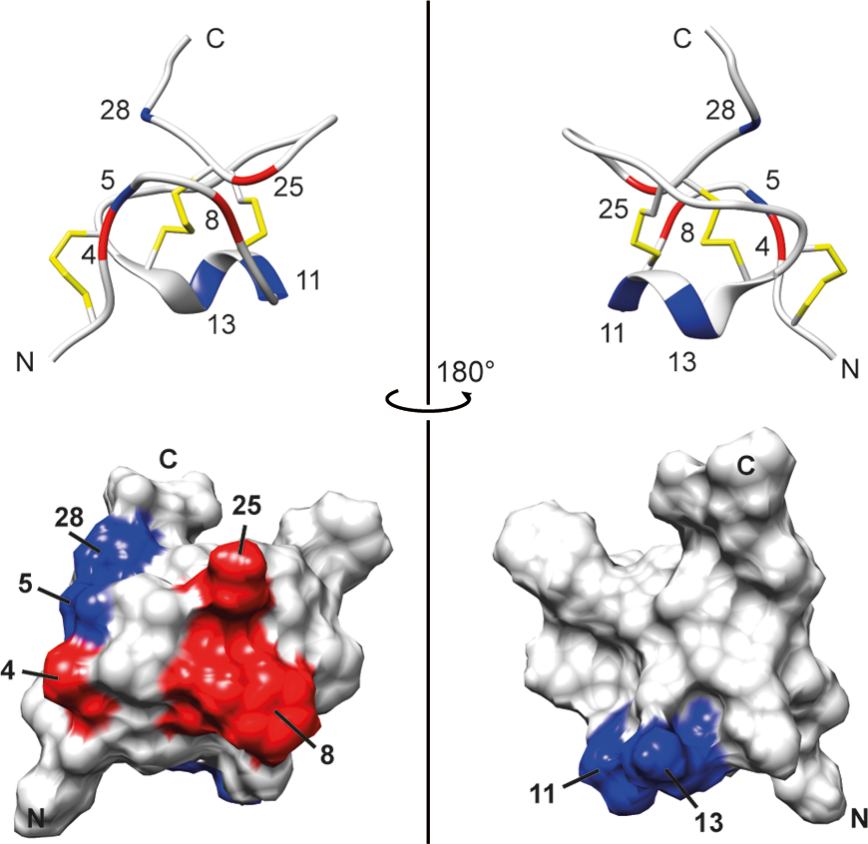
Ribbon and surface representations of **1** (RCSB pdb id: 6ENA) with the variable positions between the family members marked. Red: Positions with the highest frequency of variability: 4 (AKSP), 8 (FVGM), and 25 (KH). Blue: Positions that only differ from the archetype α sequence in α-7; positions 5 (V), 11 (I), 13 (N), and 28 (K). All red-colored positions (4, 8, 25) are situated on the same side of the molecule. Figure prepared in UCSF Chimera.^22^

In the present investigation, we verified that small differences in the amino acid (AA) sequences may have a profound effect on the peptide syntheses, folding, and biological activities. Comparing AA sequences in detail shows that the α-nemertides discovered so far vary at only seven out of 31 amino acids, positions 4, 5, 8, 11, 13, 25, and 28, as highlighted in Figure 5. Positions 4 (Ala, Lys, Ser, Pro), 5 (Thr, Val), and 8 (Phe, Val, Gly, Met) are all situated in loop 1 between Cys residues I and II. Loop 2, i.e., the sequence between Cys II and III, has two variable positions: 11 (Leu, Ile) and 13 (Lys, Asn). Loop 3 is fully conserved, whereas loop 4 contains the variable position 25 (Lys, His, and Ala of α-8). The N-terminal “loop” contains only a conserved Gly residue, whereas the C-terminal stretch contains five amino acids, one of which is the variable position 28 (Hyp, Lys). When comparing all peptides, **7** is the most divergent of the toxins, harboring seven substitutions from the consensus sequence. The pairwise alignment of **1**–**6** varies between 90.3% (**2** vs **6**) and 96.7%, whereas α**-**7 has between 77% and 80% identity, vs **1**–**4** and **5**/**6**. The most frequent variable positions (4, 8, and 25) are all positioned on the same side of the peptide, where loops 1 and 4 meet, Figure 6.

All α-nemertides tested display potent effects in *Artemia* and in Na_V_ assays. The combined results indicate grouping of peptides: **1**, **4**, and **5** showed higher activity against both *Artemia* and BgNa_V_1, compared to **2** and **3**. The *Artemia* assay clearly divides α-nemertides into two groups: the highly active **1**, **4**, and **5** (EC_50_ 0.3–0.5 μM) and moderately active toxins **2**, **3**, **6**, and **7** (EC_50_ 2.8–6.1 μM). The most active (**1**, **4**, **5**) all contain a small amino acid (Ala or Ser) in position 4 in combination with Phe8, whereas **3** and **6** with lower activity have a basic Lys in position 4. Furthermore, **2** and **3** lack an aromatic residue in position 8. Nemertide **6** shows high activity in the BgNa_V_ assay, but not in the *Artemia* microwell assay.

α-Nemertides appear to always be expressed at least in pairs on the transcriptomic level. Evidence of coexpression on the peptide level has so far only been demonstrated in *L. longissimus*, in which **1** and **2** were found to be present together in the mucus.^8^ Transcriptome analyses indicate that **1** is present together with **2** in both *L. longissimus* and *L. ruber*. *L. ruber* also expresses **7**. In *L. lacteus*, **1** is paired with **3**. The results from the assays reveal that these “pairs” are composed of toxins of different potency. For example, **1** is one order of magnitude more potent both in the *Artemia* assay and at the BgNa_V_ channel as compared to **2** and **3**. The same pattern can be observed in other pairs: the highly potent **5** occurs together with the less potent **3** in *R. pseudolacteus*, and in the case of *L. sanguineus*, **4** is paired with **6**. Both of these toxins are approximately equipotent at BgNa_V_1; **4** is however one order of magnitude more active in *Artemia*. Nemertide α-7 (**7**) found in the *L. ruber* transcriptome was the least active peptide in *Artemia*. It does however exhibit similar activity in BgNa_V_1 as compared to α-1 (**1**). The pairwise expression of α-nemertides with differing activity profiles suggests that these peptides may target different prey or predators.

Most of the α-nemertides display higher activity on the cockroach BgNa_V_1 channel than on the mammalian subtypes tested, which becomes more evident when comparing potency ratios (see Table 2 and the normalized values in Supplementary Table 1). This is especially pronounced for **1**, where the EC_50_ for BgNa_V_1 is at least nine times lower than in the other channels tested. In contrast, **3** displays virtually no difference in EC_50_ between the different Na_V_s including BgNa_V_1. The results suggest a possibility to tailor sequences to achieve a more specific selectivity profile, and some initial conclusions may be drawn from the native sequence variations in combination with the activity profiles. For example, **1** and **2** differ in position 8 only, containing Phe and Val, respectively, which makes up part of the aromatic patch. The difference is clear in the *Artemia* assay: **1** is approximately 10 times more active, demonstrating the importance of a hydrophobic/aromatic residue. Also in the Na_V_ screening, clear differences are evident; the change from Phe to Val causes loss in activity by at least 1 order of magnitude in the following Na_V_ subtypes: BgNa_V_1, Na_V_1.4, Na_V_1.6, and Na_V_1.7. A 3-fold increase in activity is observed for Na_V_1.2, while no difference is apparent for Na_V_1.1, Na_V_1.3, and Na_V_1.5.

At position 4, an increase in activity is observed when Ala (as in **1**) is exchanged to Ser (as in **4**) for Na_V_1.3, Na_V_1.4, and Na_V_1.5, while almost no differences are observed for the remaining subtypes. The influence of a basic Lys in that position (as in **6**) is deducible in the comparison between **5** (Ala4, His25) and **6** (Lys4, His25). A Lys residue at position 4 results in a loss in activity of 1 order of magnitude in the *Artemia* assay. In the Na_V_ assays, it results in a strong increase in activity at Na_V_1.1 and Na_V_1.2. Interestingly, a loss in activity by 1 order of magnitude is observed for Na_V_1.3, while almost no change in activity is observed for the remaining channels, including BgNa_V_1. Position 25 (consensus, **1**, Lys) can be compared to **5** (His), with a pronounced increase in activity for Na_V_1.3 and Na_V_1.4. In Na_V_1.6 a 4-fold increase in activity is observed in favor of His25, while almost no difference is observed for BgNa_V_1, Na_V_1.1, Na_V_1.2, Na_V_1.5, and Na_V_1.7.

### Use and Role of α-Nemertides

Do any of these compounds appear to be possible hits for further studies of applications in medicine or agriculture? Among the Na_V_ channels, Na_V_1.7 has been in particular focus for pain control.^23,24^ Neither of the nemertides tested here display especially remarkable activity nor high selectivity against this channel subtype. Na_V_1.3 is also of interest for pain control; here **5** shows high activity (9.4 nM). Interestingly, this peptide is 10-fold less active on Na_V_1.5, which may be important, as unwanted effects on this ion channel are of special concern because Na_V_1.5 is predominantly expressed in heart muscle. Notable is also the effect of **6**, as Na_V_1.1 is a target in epilepsy.^25^ Nemertide **6** is also the most favorable peptide with reference to activity on Nav1.5. However, the notorious Nav1.5 may now emerge as a potential drug target. Recently, Nijak et al. showed that nemertide **1** was able to restore the loss of function by reducing channel inactivation by binding to Na_V_1.5 in a rare inherited cardiac arrhythmia.^26^ Nemertide **4**, which normally would be immediately disqualified as the most toxic peptide because it is the most active on Na_V_1.5, might thus be the most interesting one.

Judged from activity *in vivo*, **1** still appears as one of the most promising insecticidal peptides, but in the BgNa_V_1 assay **6** again stands out as the most active compound. This leads to another question: why are the effects of some peptides predictable between *in vitro* and *in vivo*, whereas others are not? Nemertide **6** contains a Lys at position 4, adding an extra positive charge to the molecule. This could possibly indicate a bioavailability threshold. Lys4 can also be found in **3**, which has relatively low activity both in *Artemia* and at the BgNa_V_1 channel. Nemertides **2** and **3**, with low activity in both *Artemia* and BgNa_V_1, both lack a Phe in position 8, indicating an important position for insect/crustacean activity. Together, the combined results from **2**, **3**, and **6** indicate that a Lys in position 4 mainly affects the bioavailability in *Artemia*, while a Phe at position 8 is important for the actual effect at the channel.

The role of these peptides in nature is still unclear. Most ion channel toxins are described as parts of venoms,^27^ but the roles of nemertean toxins can be dual. All five species for which full-sequence α-nemertides have been identified also express both cytolysins/parborlysins and neurotoxin B/β-homologues on the transcriptomic level.^7,8^ All three toxin types have been identified in the secreted mucus and may be part of the same defense/predatory system, where the cytolysins/parborlysins may facilitate uptake of the neurotoxins into the intended prey/aggressor or be part of the protection against malevolent microorganisms.

## Experimental Section

### Synthesis of α-Nemertides **1**–**7**

Nemertides α-1–6 were assembled in 0.1 mM scale using Fmoc-chemistry-based automated SPPS essentially according to our previously described method for the synthesis and cleavage of α-1,^8^ with the following differences: for **3**–**6**, 2-chlorotrityl resin was utilized instead of the high-swelling HMPA resin previously used. Nemertides α-3–6 were assembled on 2-chlorotrityl in 0.1 mmol scale, with the first four amino acids (positions 31–28) coupled manually. The first amino acid (Gln) was loaded at 0.7 equiv using DIPEA as coupling reagent, and the unoccupied binding sites were capped using MeOH. The following Asn residue (5 equiv) was double-coupled with DIC (10 equiv) and OxymaPure (10 equiv). All hydroxyprolines were double-coupled using 3 equiv of amino acid. The resin was then transferred to a microwave peptide synthesizer (Liberty 1, CEM Corp., Matthews, NC, USA) for the remaining coupling cycles except a manually introduced dipeptide at positions 11 and 12 (Leu-Ser; using 2 equiv). Peptides were cleaved off the resin by the addition of TFA/H_2_O/TIPS (95:2.5:2.5), precipitated in ice-cold ether, redissolved in MeCN/H_2_O (1:1), lyophilized, and purified using RP-HPLC. Nemertide α**-**7 was purchased from GenScript in reduced form.

All peptides were oxidatively folded in batches of 20–25 mg: peptides were dissolved in a total of 6 mL MQ-water added in small portions with rigorous vortexing in between. DMSO (2 mL) was added to the fully dissolved peptide, and the solution was slowly added to 92 mL of 0.54 M NH_4_HCO_3_ containing 20% DMSO, 0.5 mM GSH, and 2.6 mM GSSG, yielding a final folding buffer of 100 mL of 0.5 M NH_4_HCO_3_, 20% DMSO, 0.45 mM GSH, and 2.4 mM GSSG. The folding mixture was put on a shaking table for 15–18 h. Folding was quenched by addition of 200 mL of 0.4% TFA in MQ-water.

Purification of the folded peptides was performed on RP-HPLC in accordance with our previous work,^8^ using preparative RP-HPLC on a Jupiter C18 column (300 Å 250 × 21.2 mm 10, Phenomenex, CA, USA) run in linear gradient mode (5–95% MeCN, 0.05% TFA in 45 min, 16 mL/min). Fractions (0.8 min) were collected, and a small sample of each fraction was analyzed by direct infusion into an LCQ-Deca MS (Thermo Electron, CA, USA) to identify toxin-containing fractions. The purity of selected fractions was assessed by HPLC-UV using a Kinetex XB-C18 column (C18, 250 × 4.6 mm, 5 μ, 100 Å, Phenomenex) (**6**, **7**) or a Jupiter column (C18, 250 × 4.6 mm, 5 μ, 300 Å, Phenomenex) (**1**–**5**). High-resolution MS was measured using UPLC-QToF (Waters nanoAcquity; Micromass QToF Micro; Waters, MA, USA). Quantification of the lyophilized pure, folded peptides was performed using IR spectroscopy (DirectDetect, Millipore Corp., MA, USA).

### Microwell *Artemia* Bioassay

The *Artemia* microwell assay was previously described.^21^ In short, *Artemia* cysts (Artemio pur, JBL, Neuhofen, Germany) were hatched in a separation funnel containing artificial seawater (33 g salt/L, Coral pro salt, Red Sea, Eilat, Israel) prepared in deionized water. A small aquarium air pump was used to aerate the water until the shrimp were harvested (24 h, RT).

Aliquots of 100 μL (0.003–300 μM nemertide in MQ-water, control: 100 μL of MQ-water) were added in duplicates to flat-bottom 96-well plates (cat. no.: nunc 260895, Thermo Fisher Scientific, Waltham, MA, USA). *Artemia* nauplii, 10–15 in 100 μL of salt water, were transferred to the wells and incubated at RT in the dark for 24 h. Wells were then examined under a microscope, and all dead and immobilized nauplii were counted. The surviving nauplii were sacrificed by addition of 100 μL of MeOH, followed by 15 min of incubation. The total numbers of nauplii were subsequently counted. Toxicity was calculated as % dead or immobilized/total nauplii in each well. Results were plotted in GraphPad prism 8. EC_50_ values were calculated using nonlinear regression.

### Electrophysiology on Selected Na_V_s

For the expression of Na_V_ channels (hNav1.1, rNa_V_1.2, rNa_V_1.3, rNa_V_1.4, hNa_V_1.5, mNa_V_1.6, hNav1.7, rNa_V_1.8, the insect channel BgNa_V_1, the auxiliary subunits rβ1, hβ1, and TipE) in *Xenopus laevis* oocytes, the linearized plasmids were transcribed using the T7 or SP6 mMESSAGE-mMACHINE transcription kit (Ambion, Carlsbad, CA, USA). The harvesting of stage V and VI oocytes from an anaesthetized female *X. laevis* frog was described previously.^28^ Oocytes were injected with 50 nL of cRNA at a concentration of 1 ng/nL using a microinjector (Drummond Scientific, Broomall, PA, USA). The oocytes were incubated in a solution containing 96 mM NaCl, 2 mM KCl, 1.8 mM CaCl_2_, 2 mM MgCl_2_, and 5 mM HEPES (pH 7.4), supplemented with 50 mg/L gentamycin sulfate.

Two-electrode voltage-clamp recordings were performed at room temperature (18–22 °C) using a Geneclamp 500 amplifier (Molecular Devices, Downingtown, PA, USA) controlled by a pClamp data acquisition system (Axon Instruments, Union City, CA, USA). Whole cell currents from oocytes were recorded 1–4 days after injection. The bath solution composition was 96 mM NaCl, 2 mM KCl, 1.8 mM CaCl_2_, 2 mM MgCl_2_, and 5 mM HEPES (pH 7.4), 5 (pH 7.4). Voltage and current electrodes were filled with 3 M KCl. Resistances of both electrodes were kept between 0.8 and 1.5 MΩ. The elicited currents were filtered at 1 kHz and sampled at 20 kHz using a four-pole low-pass Bessel filter. Leak subtraction was performed using a −P/4 protocol. For the electrophysiological analysis of toxins, a number of protocols were applied from a holding potential of −90 mV with a start-to-start interval of 0.2 Hz. Sodium current traces were evoked by 100 ms depolarizations to *V*_max_ (the voltage corresponding to maximum sodium current in control conditions). To assess the concentration–response relationships, data were fitted with the Hill equation: *y* = 100/[1 + (EC_50_/[toxin])^*h*^], where *y* is the amplitude of the toxin-induced effect, EC_50_ is the toxin concentration at half-maximal efficacy, [toxin] is the toxin concentration, and *h* is the Hill coefficient. All data were tested for normality using a D’Agustino Pearson omnibus normality test and for variance using Bonferroni’s test or Dunn’s test. Data following a Gaussian distribution were analyzed for significance using one-way ANOVA. Nonparametric data were analyzed for significance using the Kruskal–Wallis test. Differences were considered significant if the probability that their difference stemmed from chance was below 5% (*p* < 0.05). All data were analyzed using pClamp Clampfit 10.0 (Molecular Devices) and Origin 7.5 software (Originlab, Northampton, MA, USA).

The use of the frogs was in accordance with license number LA1210239 of the Laboratory of Toxicology & Pharmacology, University of Leuven. All animal care and experimental procedures were in accordance with the guidelines of “European convention for the protection of vertebrate animals used for experimental and other scientific purposes” (Strasbourg, 18.III.1986).

## Supporting Information Available

Figure of the purity of nemertide α toxins 1–7; table of BgNav1 normalized activity of nemertide α toxins (PDF)
