# Supplementary material for: Functional Characterization of the Nemertide α Family of Peptide Toxins
Source: J Nat Prod. 2021 Aug 16;84(8):2121–8. doi: 10.1021/acs.jnatprod.1c00104 (PMC8406415; doi:10.1021/acs.jnatprod.1c00104)
Supplement: Supplementary file 1 — np1c00104_si_001.pdf [file np1c00104_si_001.pdf]

SUPPORTING INFORMATION FOR

FUNCTIONAL CHARACTERIZATION OF THE NEMERTIDE α FAMILY OF PEPTIDE TOXINS

Erik Jacobsson[†*], *Steve Peigneur*[‡*], *Håkan S. Andersson*[†,,§], *Quentin Laborde*[†], *Malin Strand*[^],

Jan Tytgat[‡] *and Ulf Göransson*[†].

† Pharmacognosy, Department of Pharmaceutical Biosciences, Biomedical Center, Uppsala University, Box 591, SE-751 24, Uppsala, Sweden. ‡ Toxicology & Pharmacology, University of Leuven (KU Leuven), O&N 2, PO Box 992, Herestraat 49, 3000, Leuven, Belgium. § Department of Medical Biochemistry and Biophysics, Karolinska Institutet, 17177 Stockholm, Sweden. ^ Swedish Species Information Centre, Swedish University of Agricultural Sciences, 75007 Uppsala, Sweden.

* These authors contributed equally.

(ulf.goransson@farmbio.uu.se)

TABLE OF CONTENTS Page

Figure S1. Purity of Nemertide α toxins **1-7** 3

Table S1. BgNav1 normalized activity of nemertide α toxins 4

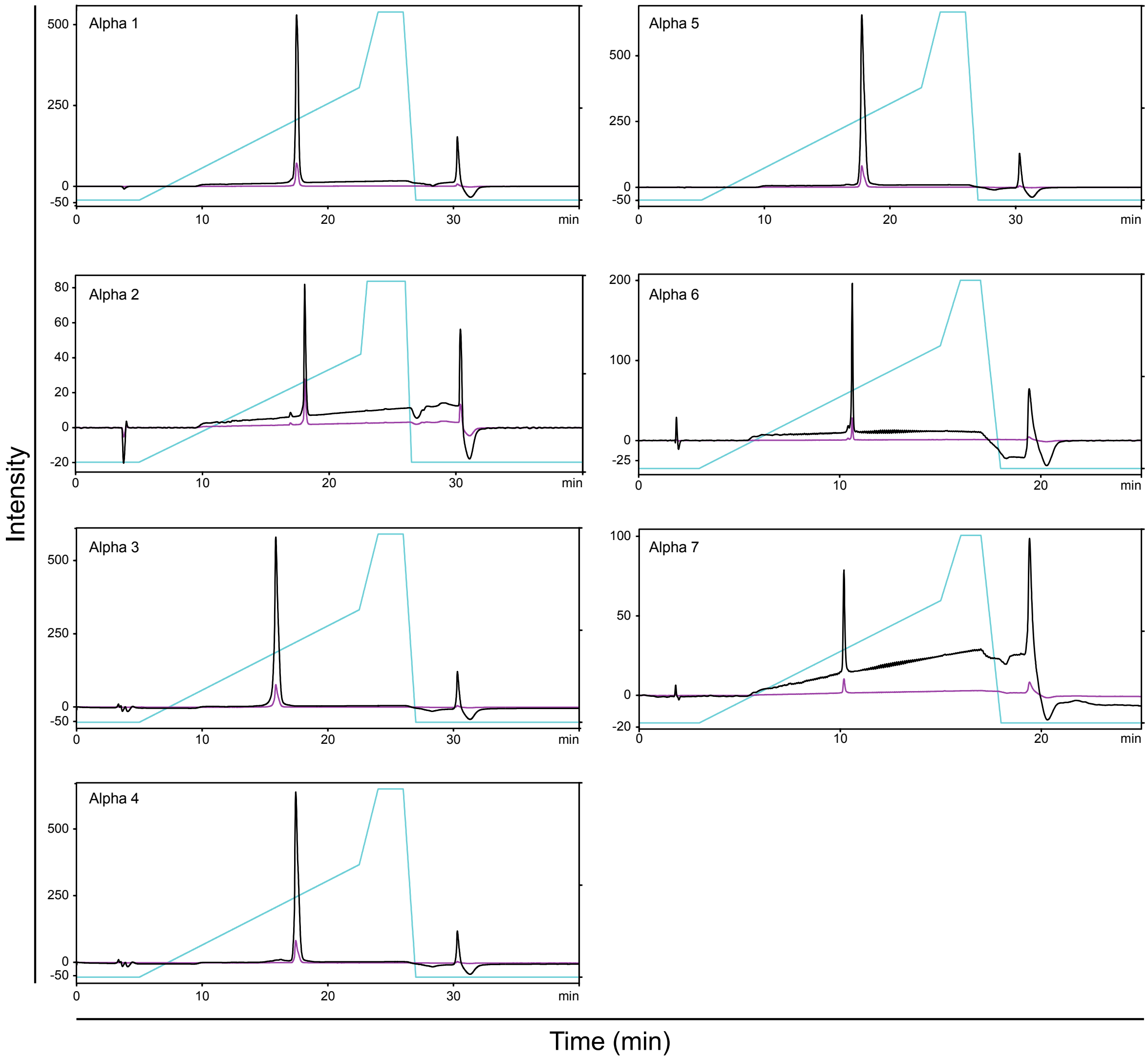

Figure S1. Purity of nemertide α toxins 1 - 7. Absorbance at 215 nm in black and absorbance at 280 nm in magenta. MeCN gradient in light blue. Note that several systems were used for the purity determination (**1-5** on Jupiter C18, **6-7** on Kinetex XB-C18), and that the retention times cannot be compared directly between samples.

Table S1. BgNav1 normalized activity of nemertide α toxins.

Nemertide	$BgNa_v1$*	$BgNa_v1$**	Nav1.1	Nav1.2	Nav1.3	Nav1.4	Nav1.5	Nav1.6	Nav1.7	Nav1.8
α1	8.6 ± 2.9	1 (3.3)	14.4	41.8	15.7	16.9	16.1	28.0	8.9	n.a.
α2	87.2 ± 10.5	1 (33.5)	1.4	1.1	1.5	13.2	1.7	15.6	14.9	n.a.
α3	97.5 ± 15.6	1 (37.5)	1.3	1.4	1.4	1.5	1.1	1.0	1.0	n.a.
α4	11.1 ± 1.6	1 (4.3)	8.3	12.1	1.2	1.3	2.5	11.1	7.3	n.a.
α5	7.8 ± 3,2	1 (3.0)	13.1	20.0	1.2	2.0	17.0	8.6	9.4	n.a.
α6	2.6 ± 0.3	1 (1.0)	3.0	9.3	40.6	17.8	82.8	14.0	37.4	n.a.
α7	9.5 ± 1.2	1 (3.7)	18.1	5.3	17.9	85.3	16.4	15.5	13.6	n.a.

* non-normalized values (nM). ** relative activity to the most $BgNa_V1$ active nemertide (α **6**) in brackets. n.a.: not active.
